# Assessment of risk factors associated with outpatient parenteral antimicrobial therapy complications

**DOI:** 10.1017/ash.2022.87

**Published:** 2022-05-16

**Authors:** Christina Kaul, Jenny Yang, Matthew Haller, Sadie Solomon, Yaojie Wang, Rong Wu, Yu Meng, Robert Pitts, Michael Phillips

## Abstract

**Background:** Outpatient parenteral antimicrobial therapy (OPAT) is used in the outpatient setting to treat infectious conditions that require a prolonged course of antimicrobials. OPAT has been shown to decrease length of hospital stay and healthcare costs without compromising patient care and has become a widely accepted practice nationally. Due to this trend, the study of OPAT is of vital importance and will continue to be relevant moving forward. Currently, few studies have explored risk factors associated with OPAT complications, and most are limited in their analysis by indication. Further work should be performed to expand upon what is currently known. We characterized factors associated with increased OPAT complication risk. **Methods:** We conducted a retrospective cohort study at 4 sites across NYU Langone Health in patients admitted from 2017 to 2020. We applied the following inclusion criteria: aged ≥18 years and discharged with OPAT. Complications were defined as follows: vascular-access-related (line occlusion, thrombosis, dislodgement, central-line associated bloodstream infection or CLABSI) and antimicrobial-related (laboratory derangement, drug reaction, *Clostridioides difficile* infection), all-cause 30-day readmission, and OPAT-related readmission. Data were obtained from electronic medical records and the OPAT database. This study was granted a waiver from informed consent by the NYU Institutional Review Board. Multivariate logistic regression was performed, adjusting for confounding variables (sex, age, hospital of admission, history of chronic medical conditions, line type, and line duration). **Results:** Overall, 1,846 patient encounters of 5,951 reviewed met inclusion criteria. The median age was 66 (IQR, 26), 42.2% were female. Moreover, 810 (44%) received a peripherally inserted central catheter (PICC) and 1,036 (56%) received a midline cathether. Also, 563 (30.5%) were discharged to subacute rehabilitation (SAR). The most frequent complications were line dislodgement (4.2% of all patients), laboratory derangement (3.0%), and drug reaction (2.4%). Furthermore, 27 patients (1.5%) developed CLABSI. Patients discharged to SAR were more likely to develop CLABSI (OR, 4.1l; *P* = .005), and they had higher rates of OPAT-related 30-day readmissions (OR, 2.675; *P* = .004) compared to those who were discharged home, after adjusting for key confounders. **Conclusions:** Discharge to SAR is strongly associated with increased risk of readmission for OPAT-related complications and CLABSI, after adjusting for key confounders. CLABSI prevention during SAR admission is a critically needed public health intervention.

**Funding:** None

**Disclosures:** None

